# Associations of adversity in childhood and risk factors for cardiovascular disease in mid-adulthood

**DOI:** 10.1016/j.chiabu.2017.10.015

**Published:** 2017-11-02

**Authors:** Emma L. Anderson, Abigail Fraser, Rishi Caleyachetty, Rebecca Hardy, Debbie A. Lawlor, Laura D. Howe

**Affiliations:** a MRC Integrative Epidemiology Unit at the University of Bristol, Bristol, UK; b School of Social and Community Medicine, University of Bristol, Bristol, UK; c The Institute of Applied Health Research, University of Birmingham, UK; d MRC Unit for Lifelong Health and Ageing at University College London, UK

**Keywords:** Psychosocial, Adversity, Childhood, Cardiovascular disease

## Abstract

Studies assessing associations of childhood psychosocial adversity (e.g. sexual abuse, physical neglect, parental death), as opposed to socioeconomic adversity, with cardiovascular disease (CVD) risk factors in adulthood are scarce. The aim of this study is to assess associations of various types of psychosocial adversity and cumulative adversity in childhood, with multiple CVD risk factors in mid-life. At study enrolment, women from the Avon Longitudinal Study of Parents and Children (N = 3612) retrospectively reported: lack of maternal care, maternal overprotection, parental mental illness, household dysfunction, sexual abuse, physical and emotional abuse, and neglect in childhood. Approximately 23 years later, body mass index (BMI), waist circumference, systolic and diastolic blood pressure, plasma glucose, insulin, triglycerides, low and high density lipoprotein cholesterol, C-reactive protein, carotid intima-media thickness (cIMT) and arterial distensibility were assessed (mean age 51 years). We examined associations of each specific type of psychosocial adversity and cumulative adversity with CVD risk factors. No specific type of psychosocial adversity was consistently associated with the CVD risk factors. There was evidence that a one standard deviation greater cumulative psychosocial adversity was associated with 0.51 cm greater waist circumference (95% confidence interval [CI]: 0.02 cm, 1.00 cm, p = 0.04) and a lower arterial distensibility, even after adjustment for age, ethnicity and childhood and adult socioeconomic position. We found no consistent evidence that any specific type of psychosocial adversity, or cumulative psychosocial adversity in childhood, is associated with CVD risk factors in adult women.

## 1. Introduction

Studying the long-term health consequences of adversity in childhood is important for providing evidence to support the potential benefits of public interventions that seek to prevent adversity in childhood. The American Heart Association recently published a statement to increase awareness of the influence of social factors on the incidence, treatment, and outcomes of CVD. They recommended further observational studies examine the complex interactions between social factors and ardiovascular health (Bayer, Fairchild, Hopper, & Nathanson, 2013). There is now consistent evidence showing that socioeconomic adversity in childhood (e.g. low head of household social class, household overcrowding and low parental education) is associated with cardiovascular disease (CVD) risk factors in adulthood (Galobardes, Smith, & Lynch, 2006; Havranek et al., 2015). However, there is much less evidence for associations of psychosocial adversity in childhood (for example, sexual abuse and physical neglect) with later CVD risk, and existing studies have reported both positive and null findings (Crowell et al., 2015; Gooding et al., 2016; Halonen et al., 2015; Hosang et al., 2013; Rich-Edwards et al., 2012; Su et al., 2015; Thurston et al., 2014). The Adverse Childhood Experiences (ACE) study has been pivotal in characterising medium and long-term health outcomes of psychosocial adversity in childhood and Felitti et al. have previously reported a strong graded relationship between the number of categories of retrospectively reported adverse childhood exposures and the presence of CVD (including ischemic heart disease) in a study of 9508 adults (Felitti et al., 1998).

Existing studies have reported associations between specific types of psychosocial adversity, particularly sexual or physical abuse, (Gooding et al., 2016; Hosang et al., 2013; Rich-Edwards et al., 2012; Thurston et al., 2014) with increased CVD risk. However, few studies have considered a possible cumulative effect of exposure to multiple types of adversity in childhood (Crowell et al., 2015; Halonen et al., 2015; Su et al., 2015). Assessing the effects of cumulative psychosocial adversity acknowledges that adverse experiences tend to co-occur and that experiencing multiple types of psychosocial adversity in childhood may have a greater adverse effect on cardiovascular health than experiencing only one. Furthermore, there is some evidence to suggest that associations between childhood adversity and CVD risk in adulthood may differ between people who have high compared to low socioeconomic position (SEP) in adulthood (Halonen et al., 2015).

We aimed to assess associations of maternal lack of care, maternal overprotection, parental mental illness, household dysfunction, sexual abuse and physical or emotional abuse or neglect in childhood (each individually), and cumulative psychosocial adversity, with several established risk factors for cardiovascular disease including body mass index (BMI), waist circumference, systolic and diastolic blood pressure (SBP and DBP), plasma glucose, insulin, triglycerides, low and high density lipoprotein cholesterol (LDL and HDL), C-reactive protein (CRP), carotid intima media thickness (cIMT) and arterial distensibility, in adult women (Pearson et al., 2002; Pearson et al., 2003; Tehrani & Wong, 2015). Greater levels of each of these risk factors have been previously associated with higher risk of diabetes (Janiszewski, Janssen, & Ross, 2007; Mohammadifard et al., 2013), hypertension (Shihab et al., 2012), ischemic stroke (Rost et al., 2001), ischemic heart disease (Jeppesen, Hein, Suadicani, & Gyntelberg, 1998, Jeppesen, Hein, Suadicani, & Gyntelberg, 2000) and coronary heart disease (Flint et al., 2010), except for HDL whereby greater levels have been associated with lower risk of CVD (Rader & Hovingh, 2014). We also examined whether (i) observed associations were independent of SEP in childhood, (ii) whether they were mediated by SEP, BMI and smoking in adulthood, and (iii) whether they differed in women with high compared with low SEP in adulthood.

## 2. Methods

### 2.1. Study populations

**Fig. 1:**
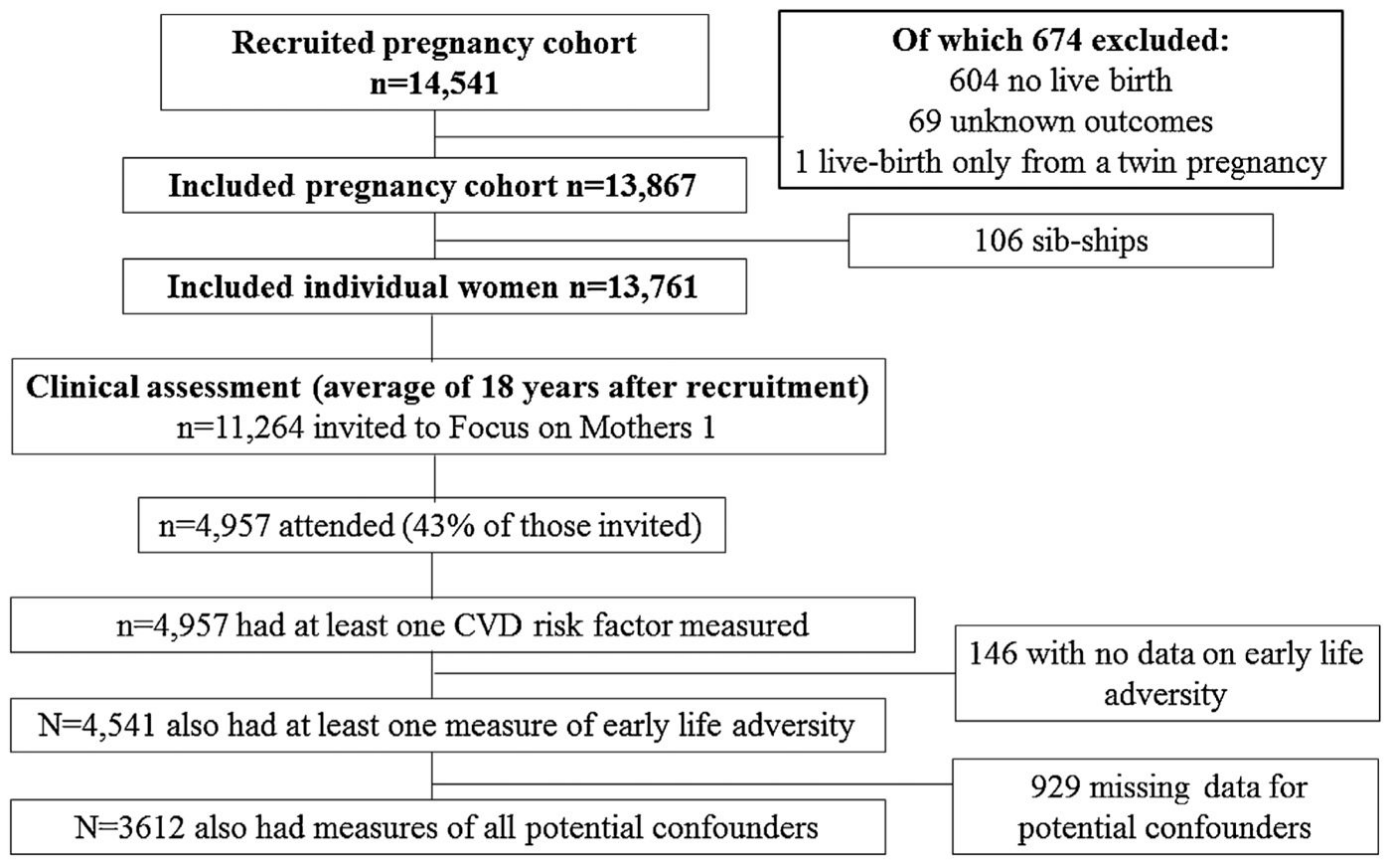
Participant flow through the study.

The Avon Longitudinal Study of Parents and Children (ALSPAC) is a prospective birth cohort study from southwest England (full details in online supplement)(Boyd et al., 2012; Fraser et al., 2012). The study website contains details of all available data through a fully searchable data dictionary (www.bris.ac.uk/alspac/researchers/data-access/data-dictionary/). Ethical approval for the study was obtained from the ALSPAC Ethics and Law Committee and the Local Research Ethics Committees. Briefly, ALSPAC recruited 14 541 pregnant women with expected delivery dates between April 1, 1991 and December 31, 1992. Approximately 18 years after recruitment into the cohort (mean age 51 years), 4957 women attended a follow-up research clinic at which CVD risk factors were assessed (Fig. 1).

### 2.2. Assessing CVD risk factors

The following CVD risk factors were assessed at a research clinic at mean age 51 years: BMI; waist circumference; SBP, DBP; fasting plasma glucose, insulin, triglycerides, LDL, HDL and CRP; cIMT and arterial distensibility. Full assessment details of each CVD risk factor are provided in the online supplement.

### 2.3. Assessing adverse experiences in childhood

Exposure to psychosocial adversity in childhood (before age 17 years) was retrospectively reported in questionnaires administered at the time of enrolment into the study, throughout pregnancy and postnatally (from 12 weeks gestation to 33 months postnatally). *A priori,* we aimed to examine the same adversity measures as the Adverse Childhood Experiences (ACE) study (Felitti et al., 1998). However, ALSPAC measured many additional forms of adversity to this study. Thus, we decided to include as many types of psychosocial adversity as possible.

The following types of psychosocial adversity were considered: maternal lack of care, maternal overprotection, parental mental illness, household dysfunction, sexual abuse, and non-sexual abuse (physical and emotional abuse or neglect). Questions about maternal care and overprotection were based on a validated instrument for assessing maternal bonding (Parker, 1990). Maladaptive family functioning includes questions that assess the nature of the relationship between the participant’s mother and father (i.e. whether the relationship was, for example, stable and predictable, affectionate, violent, respectful). Parental mental illness includes questions about depression, anxiety, schizophrenia or alcoholism in the participant’s mother or father. Sexual abuse questions assessed experiences of various types of sexual abuse by different people (e.g. family members, friends or strangers). Non-sexual abuse includes questions that capture physical or emotional cruelty and neglect by either parent/guardian. It is important to note that although there may appear to be overlap between ‘maternal lack of care’ and ‘emotional cruelty or neglect’, the questions assessing the latter reflect neglect by either parent/guardian; not just the mother. Details of the exact questions asked about each type of psychosocial adversity are provided in the online supplement.

### 2.4. Assessment of covariables

Women reported their father’s occupation during childhood (i.e. childhood SEP), retrospectively in questionnaires administered during pregnancy. Responses were used to allocate head of household social class groups, coded as ‘manual’ or ‘non-manual’, based on the Standard Occupational Classification 2000. Participants’ SEP in adulthood was reported as the highest of own and partner’s occupational social class using the 1991 British Office of Population and Census Statistics (OPCS) classification. Unlike childhood SEP, adult SEP could not be coded as ‘manual’ and ‘non-manual’ due to the low prevalence of ‘manual’ social class in this sample. Thus, it was coded as ‘high’ (professional, managerial and technical occupations) or ‘low’ (including skilled, partly skilled and unskilled occupations). Women reported their ethnicity, pre-pregnancy weight and height (from which BMI was calculated as weight in kilograms divided by height in meters-squared) and whether they were a current smoker, ex-smoker or had never smoked, in questionnaires administered at enrolment. Women reported their menopausal status at mean age 50 years (relevant for sensitivity analysis examining whether our observed findings could be largely explained by menopausal status; full details in the statistical analysis section below). Age was recorded at the time of outcome assessment along with details of current medication use.

## 3. Statistical analysis

### 3.1. Associations of specific types of psychosocial adversity in childhood and CVD risk factors

**Fig. 2:**
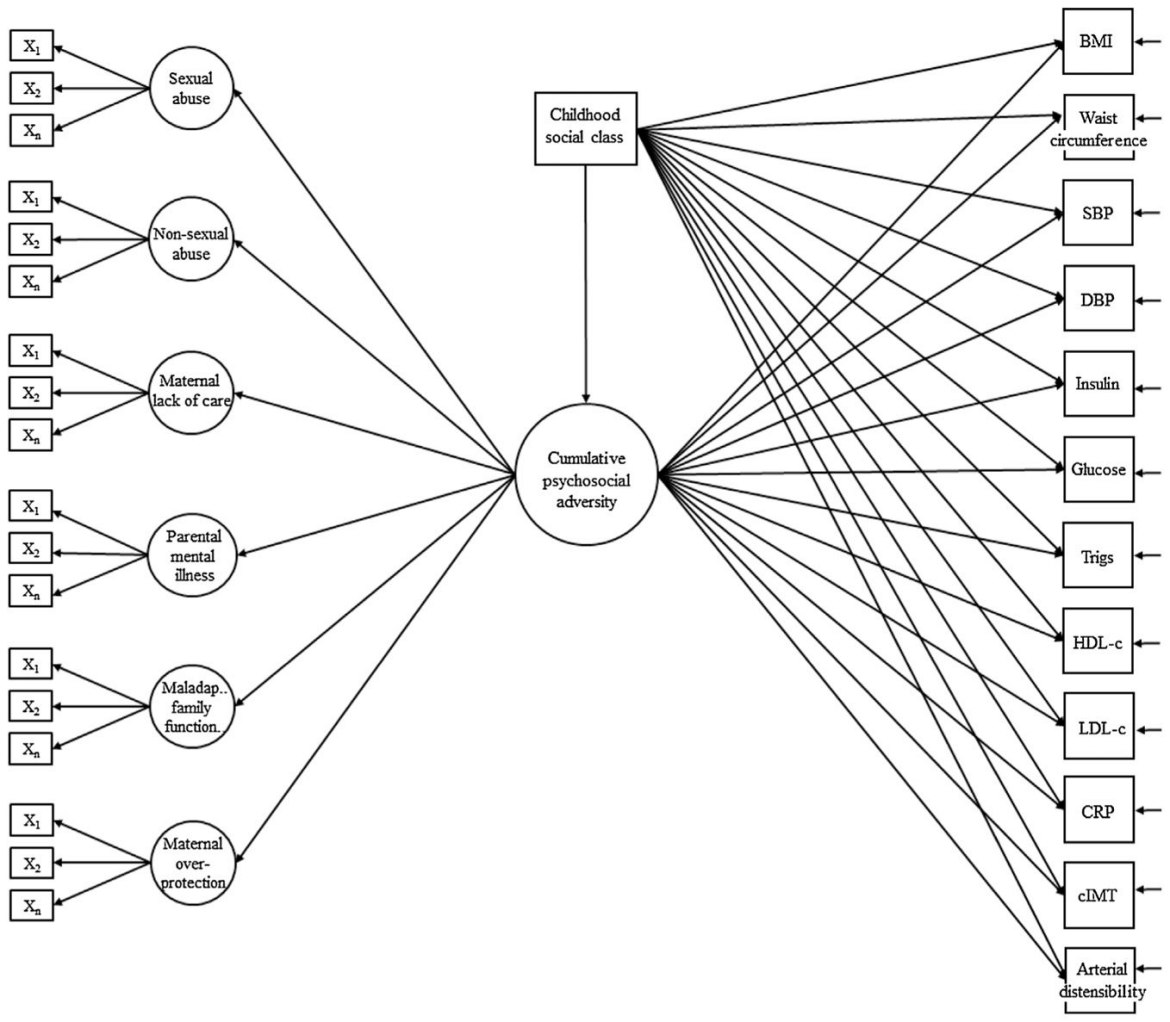
Structural equation model. X_1-n_ represent observed binary indicators that load onto each single latent psychosocial adversity construct (i.e. the first order factors). A complete list of the binary indicators for each latent construct is provided in Supplemental Table S1, along with their factor loadings and model fit statistics. Cumulative psychosocial adversity is a second order factor that captures each of the single latent psychosocial adversity constructs. Supplemental Table S1 provides factor loadings for each of the first order factors onto the second order factor. Childhood social class has been shown as a covariate for illustrative purposes; in our confounder adjusted model, ethnicity and age at outcome assessment were additionally adjusted for. CVD risk factors are multivariate outcomes with residual covariances.

As there were multiple questions assessing each specific type of adversity, we first sought to combine all available questions into single variables. Thus, we used confirmatory factor analysis to create single latent constructs for maternal lack of care, maternal overprotection, parental mental illness, household dysfunction, sexual abuse and non-sexual abuse (Fig. 2 and Supplemental Table S1). We then estimated a latent construct of total psychosocial adversity in childhood, which was informed by each of these single latent constructs. Latent constructs are variables that are not directly observed, but are inferred from other variables that are observed or measured (i.e. responses to the adversity questions). Higher latent trait values are indicative of greater levels of adversity. Full methods and model fit statistics for the confirmatory factor analyses are provided in the online supplement. Analyses were conducted using Mplus version 7.31 (Muthén & Muthén, 2008).

Structural equation models (Fig. 2) were used to simultaneously conduct the confirmatory factor analyses and estimate associations of each type of psychosocial adversity in childhood with the CVD risk factors in the following regression models: (1) adjusted for age at outcome assessment (2) additionally adjusted for childhood SEP and ethnicity (3) additionally adjusted for potential mediation by adult SEP and (4) additionally adjusted for potential mediation by adult BMI (except for when BMI and waist circumference were outcomes) and smoking. We also tested for an interaction between specific types of psychosocial adversity and adult SEP by using likelihood ratio tests, to compare a model in which an interaction tern for adult SEP was included to one in which adult SEP is adjusted for.

### 3.2. Associations of cumulative psychosocial adversity in childhood and CVD risk factors

Most existing studies that have assessed cumulative psychosocial adversity in childhood have used simple summary scores (i.e. totalling the number of adverse experiences) (Crowell et al., 2015; Halonen et al., 2015; Su et al., 2015). Summary scores, arguably unrealistically, assume each adverse exposure to have the same direction and magnitude of association with the outcome. We used a data-driven approach to create a cumulative psychosocial adversity score that weights each adversity exposure based on how strongly it correlates with other adversity exposures (i.e. allocating exposures that tend to co-occur with others a higher weight, so that they contribute more to the cumulative adversity score). We used confirmatory factor analysis to estimate a latent construct of cumulative psychosocial adversity in childhood, which was informed by each of the single latent constructs listed above. Higher values of this score are indicative of greater levels of psychosocial adversity in childhood. Structural equation models (Fig. 2) were used to simultaneously conduct this factor analysis and estimate associations of cumulative psychosocial adversity in childhood with the CVD risk factors, in the regression models listed above. Distributions of insulin, triglyceride and CRP were right skewed; thus, the log of these variables was used in analysis to approximate a normal distribution and regression coefficients were back transformed to ratios of geometric means, which can be interpreted as percentage difference.

### 3.3. Missing data and eligibility criteria

Missing data were dealt with using the weighted least squares means and variance adjusted (WLSMV) estimator, which assumes data are missing at random, conditional on the variables included in the model (Kivimaki, Lawlor et al., 2006). The WLSMV estimator permits the inclusion of women with incomplete data for endogenous variables. Endogenous variables are those that are predicted by other variables in our model (see Fig. 2). All variables in our structural equation model are endogenous, except for the potential confounders, which are exogenous (i.e. they are not predicted by any of the other variables in the model). The WLSMV estimator, therefore, does not handle missing data for the confounders in our model. For that reason, participants were considered eligible for inclusion if they had at least one measure of psychosocial adversity in childhood, at least one CVD risk factor and complete data for each of the potential confounders (n = 3612).

To mitigate this reduction in sample size due to only including participants with complete data for potential confounders, we conducted an additional analysis in which inverse probability weighting was used to account for participants with missing data not being included in our analysis (Mansournia & Altman, 2016). The inverse probability weighting method inflates the weights (or contributions) of participants in the regression analyses, who are under-represented due to missing data. Logistic regression models were derived to predict inclusion in our main analysis sample of women who did have complete confounder data (n = 3612), from the larger sample of women with missing data for potential confounders (n = 4541). Several strong predictors of dropout were included (e.g. baseline demographics, childhood psychosocial adversity, parental mental health, parental death and maternal education, see Table S2). If participants were missing data for these predictor variables (Table S2), they were coded as having the mode value (all were categorical variables). The inverse of the predicted probabilities was used to weight the subsequent regression analyses conducted on our main sample of 3612 women.

### 3.4. Sensitivity analyses

We assessed whether results were similar when (i) adjusting for current use of statin, antihypertensive and diabetic medication and (ii) removing participants that reported taking any of these medications at the time of outcome assessment. To investigate whether our findings were robust to missing data, we repeated analyses in participants with no missing data for any variable (i.e. a complete case analysis, n = 2101). To investigate potential selection bias, we examined associations of cumulative psychosocial adversity with a much earlier measure of BMI (at mean age 29 years as opposed to mean age 51 years). At this early stage of the study, attrition was low and therefore sample numbers are much larger (n = 8517 at mean age 29 years compared to our analysis sample of n = 3612 at mean age 51 years). As previously mentioned, there is evidence that women’s risk for CVD increases after menopause (Janssen, Powell, Crawford, Lasley, & Sutton-Tyrrell, 2008; Rossouw et al., 2007), with an increased incidence of myocardial infarction about 10 years after the onset of menopause (Hodis & Mack, 2008). Given the mean age of the study participants at outcome assessment (mean age 51 years), it is possible that the women in our study may be too young to be able to identify associations of early life adversity with CVD risk. To investigate this, we examined whether associations were similar in the subgroup of women who reported reaching menopause by the time CVD outcomes were assessed (n = 890), compared to the whole sample.

## 4. Results

### 4.1. Descriptive characteristics of the sample

Table 1 compares characteristics of women included in the current study (n = 3612) to those excluded due to missing data (n = 10,836, of which n = 1007 had CVD risk factors measured). There was evidence that included women had, on average, better values for most CVD risk factors, were older, less likely to be a current or ex-smoker, less likely to be non-white and more likely to have a non-manual childhood and adult SEP than those excluded due to missing data. There was also evidence of a higher prevalence of most of the psychosocial adversity exposures in women excluded due to missing data (Table S1 of the online supplement). However, the magnitude of each of these differences was very small. Table S3 of the online supplement gives the prevalence of each adverse psychosocial experience, by high and low childhood SEP. Women with a manual childhood SEP compared to non-manual had worse average score for household dysfunction. All other psychosocial adversity exposures were similarly distributed between manual and non-manual childhood SEP.

### 4.2. Associations of different types of psychosocial adversity in childhood and CVD risk factors

Lack of maternal care was associated with greater waist circumference, high levels of insulin and a lower arterial distensibility, however, conversely it was also weakly associated with lower CRP (Tables S4 of the online supplement). Maternal overprotection was associated with greater waist circumference, greater cIMT and a lower arterial distensibility, but was not associated with the other nine CVD risk factors.

### 4.3. Associations of cumulative psychosocial adversity in childhood and CVD risk factors

There was evidence that greater cumulative psychosocial adversity was associated with greater waist circumference and lower arterial distensibility (Table 2). These associations remained after adjustment for potential confounders and for adult SEP. After additionally adjusting for potential mediation by BMI and smoking in adulthood, the association with waist circumference attenuated to the null, but the association with arterial distensibility remained. There was no evidence that cumulative psychosocial adversity was associated with the other ten CVD risk factors. Results were very similar when using inverse probability weightings to inflate the weights of participants in the regression analyses who are under-represented due to missing data.

There was evidence of an interaction between cumulative psychosocial adversity and adult SEP for three of the twelve (25%) CVD risk factors; greater cumulative psychosocial adversity was associated with greater BMI, waist circumference and CRP in participants who had high adult SEP, whereas it was not associated with BMI, waist circumference or CRP in participants who had low adult SEP (Table S5).

### 4.4. Sensitivity analyses

Results were similar after adjusting for medication use and when removing people taking medication at the time of outcome assessment (Tables S6 and S7 of online supplement). Point estimates were also similar in the complete case analysis (with no missing data, n = 2101) and in the main analysis with multiple imputation (Table S8 of online supplement). The association of cumulative psychosocial adversity with pre-pregnancy BMI at mean age 29 years (n = n = 8517) was very similar to that with BMI at mean age 51 years (n = 3612) (Table S10). Results were also very similar in the subgroup of women who had reached menopause at the time of outcome assessment (n = 890, Table S11).

## 5. Discussion

We found no evidence to suggest that cumulative psychosocial adversity, or any specific type of psychosocial adversity, was consistently associated with CVD risk factors in adult women, before or after adjusting for childhood SEP. There was evidence to suggest associations of cumulative psychosocial adversity in childhood with only two of the twelve CVD risk factors (waist circumference and arterial distensibility), and although it is possible that psychosocial adversity in childhood is associated with some, but not all CVD risk factors, it is worth noting that there was no evidence of any specific type of psychosocial adversity being associated with either of these two outcomes.

There are a number of possible reasons for finding no evidence of associations between cumulative or specific types of psychosocial adversity in childhood and CVD risk factors in adult women. Firstly, it is possible that neither cumulative nor specific types of psychosocial adversity are causally related to CVD risk, and that socioeconomic factors and ethnicity (i.e. more broad social constructs) are more likely to be associated with these outcomes than the forms of adversity studied here. Secondly, there may have been a lack of power to detect associations due to the low prevalence of some of the psychosocial adversity exposures in this cohort. Thirdly, given all measures of psychosocial adversity in childhood were retrospectively reported during adulthood, it is possible that these measures are affected by recall bias. Thus, the lack of observed associations may, at least in part, be due to non-differential measurement error in the exposure variable, resulting in bias towards the null (Thomas, Stram, & Dwyer, 1993). That said, several other studies have reported associations between retrospectively reported adversity in childhood and CVD risk factors (Felitti et al., 1998; Halonen et al., 2015). Finally, it is possible that the effect of early life adversity on CVD risk may be masked by protective factors (e.g. if women in our study who were subject to early life adversity are subsequently subject to various protective factors such as social support, they may not have the same increase CVD risk as women who did not receive that support). These three possible explanations for the largely null associations in our study are not mutually exclusive; it is possible that there are true weak associations that are masked by lack of power and reporting bias.

There was some evidence that associations of cumulative psychosocial adversity in childhood and CVD risk factors differed in participants with low, compared to high adult SEP, but only for three of the twelve CVD risk factors. Greater cumulative psychosocial adversity in childhood was associated with greater BMI, waist circumference and CRP, but only in participants who had high adult SEP. There was no evidence for associations in participants who had low adult SEP. We would have expected these interactions to go in the opposite direction (i.e. that the association between cumulative psychosocial adversity in childhood and CVD risk only be apparent in the group with low adult SEP, or at least greater in magnitude in the group with low SEP, compared with the high). Although, it is worth noting that other studies in the same cohort of women have observed similar patterns (i.e. stronger associations between adversity in childhood and ill health in adulthood in those with high SEP) (Howe et al., 2016). The unexpected findings could possibly be due to selection bias. Loss-to-follow-up was greater in women with low adult SEP, making it plausible that low SEP participants who remain in the study are the resilient, ‘more healthy’ individuals, resulting in a lack of observed associations in this subgroup. To examine the possibility of selection bias, we compared findings across both imputed data, which, provided the data are missing at random, minimises potential for bias and complete case data (with no missing data). Point estimates were very similar across the two datasets, albeit with wider confidence intervals for the complete case analysis due to smaller sample numbers. We also compared associations of cumulative adversity with BMI at mean age 51 years, to associations of cumulative adversity with an earlier measure of BMI at mean age 29 years (where there was much less missing data) and results were very similar. Thus, it is unlikely that our null findings are fully explained by selection bias.

### 5.1. Comparison with other studies

The association between socioeconomic position in childhood and subsequent CVD risk in adulthood is now well established, with a considerable body of evidence showing markers of low socioeconomic status in childhood to be associated with CVD risk factors (Kivimaki, Smith, Juonala, et al., 2006), metabolic syndrome (Puolakka et al., 2016), vascular structure and function (Kivimaki, Lawlor, et al., 2006; Kivimaki, Smith, Elovainio, et al., 2006; Kivimaki, Smith, Juonala, et al., 2006; Puolakka et al., 2017) and risk of coronary heart disease (Lawlor, Smith, & Ebrahim, 2004). Less evidence exists for associations with psychosocial adversity in childhood. Previous studies have reported associations between specific types of psychosocial adversity in childhood and increased CVD risk;(Gooding et al., 2016; Hosang et al., 2013; Rich-Edwards et al., 2012; Thurston et al., 2014) particularly sexual and physical abuse. Very few studies have assessed associations of cumulative psychosocial adversity in childhood with multiple CVD risk factors in adulthood. In line with our findings, Bleil et al. observed no association between retrospectively reported cumulative psychosocial adversity in childhood and CVD risk in a much smaller study than ours (650 pre-menopausal adult women) (Bleil et al., 2013). Contrary to our findings, Felitti et al. reported a strong graded relationship between the number of categories of retrospectively reported adverse childhood exposures and the presence of adult diseases including ischemic heart disease, in a study of 9508 adults (Felitti et al., 1998). Halonen et al. reported that women and men (analysed together) who retrospectively reported exposure to socioeconomic and psychosocial adversity in childhood and adult neighbourhood disadvantage, had increased CVD risk, but those with only one of these exposures had little excess risk (N = 37 699) (Halonen et al., 2015). The difference between those findings and ours may, at least in part, be explained by the fact that the outcomes in those studies were not objectively assessed (most were self-reported). That said, two other recent smaller studies (N < 400) have reported associations between greater cumulative psychosocial adversity in childhood and higher blood pressure in adult men and women (Crowell et al., 2015; Su et al., 2015). and Pretty et al. found that exposure to 4 or more adverse childhood experiences (compared to fewer than 4 adverse experiences) was associated with increased BMI, waist circumference and resting heart rate in 1234 children, mean age 12 years (Pretty, O’Leary, Cairney, & Wade, 2013).

### 5.2. Strengths and limitations

Our study is relatively large and from a well characterised cohort, meaning we had data for a wider variety of psychosocial adversity exposures in childhood, and more objectively-measured CVD risk factors than previous studies. This allowed us to assess associations of several different types of psychosocial adversity in childhood as well as a comprehensive cumulative score of psychosocial adversity in childhood. Our statistical approach improves on existing studies that have either assessed associations only between separate types of psychosocial adversity and CVD risk (since this ignores co-occurrence and likely cumulative effects), or simply added up the number of adverse experiences into a summary score (since this method weights each type of psychosocial adversity equally).

One limitation of our study is the possibility of selection bias due to loss-to-follow-up, as outcomes were assessed approximately 18 years after recruitment into the cohort. The sample included in this current analysis represents approximately 25% of the original ALSPAC mothers’ cohort. However, our sensitivity analyses (discussed above) did not indicate that selection bias is likely to fully explain our results. Women’s risk for cardiovascular disease increases after menopause, with an increased incidence of myocardial infarction about 10 years after the onset of menopause (Hodis & Mack, 2008). Thus, it is possible that the women in our study were too young to be able to identify associations of early life adversity with CVD risk. To investigate this, we examined associations in the subgroup of women who reported reaching menopause by the time CVD outcomes were assessed, and results were very similar to the main analyses, suggesting that menopausal status does not fully explain our largely null findings. We only studied women, who are largely of European origin, thus we cannot assume that our results would generalise to men or women from different ethnic backgrounds. Moreover, we only assessed women who are mothers’ and it is possible that this is itself may confer protection against the effects of psychosocial adversity in childhood. All psychosocial adversity data were retrospectively self-reported in adulthood meaning there is potential for recall bias. That said, there is no gold standard method for collecting data on adverse experiences in childhood and prospective parental or self-reporting during childhood may be equally biased. A review examining the validity of adult retrospective reports of adverse childhood experiences concluded that retrospective recall in adult life of exposure to adverse experiences in childhood is sufficiently valid (Hardt & Rutter, 2004). Reverse causation is extremely unlikely in this study; childhood adversity was retrospectively reported an average of 23 years prior to the assessment of CVD risk factors. Thus, we are able to draw some conclusions about temporality of events because CVD risk factors at average age 51 years is extremely unlikely to affect (1) whether participants experienced psychosocial adversity (such as sexual abuse or parental divorce) during childhood and (2) whether participants accurately reported experiencing adversity in childhood, 23 years prior to the CVD risk factor assessment. Finally, our mediation analysis assumes no measurement error in the mediator and, given our single measure of adult SEP (occupational social class), we are unable to rule this out.

## 6. Conclusions

We found no evidence to suggest that cumulative psychosocial adversity in childhood, or any specific type of psychosocial adversity in childhood, are associated with CVD risk factors in adult women of largely European ethnic origin. Our findings suggest that any interventions put into place to support children who experience adversity are unlikely to influence CVD risk, but that is not to say they would not confer benefits elsewhere (for example, improved mental health outcomes). Thus, they do not remove the need to support those who experience adversity in childhood. Further large, well characterised studies are needed to ascertain whether associations between cumulative psychosocial adversity and CVD risk factors are different in those with high compared to low adult SEP.

## Supplementary Material

Supplement

## Figures and Tables

**Table 1:** Characteristics of participants.

	Included participants (n = 3612)		Excluded participants* (n = 10,929)		
		
	N with available data	Mean (SD)/Median (IQR)/Percentage (%)	N with available data	Mean (SD)/Median (IQR)/Percentage (%)	P for difference
Latent psychosocial adversity scores					
Sexual abuse score (SD scores)	3378	0.17 (0.54)	–	–	–
Non-sexual abuse score (SD scores)					
Parental mental illness score (SD scores)	3612	0.16 (0.54)	–	–	–
Household dysfunction score (SD scores)	3613	0.10 (0.78)	–	–	–
Maternal lack of care score (SD scores)	3550	0.08 (0.83)	–	–	–
Maternal overprotection score (SD scores)	3549	< 0.012 (0.78)	–	–	–
Cumulative psychosocial adversity score (SD scores)	3612	0.14 (0.77)	–	–	–
Outcomes					
BMI (kg/m^2^)	3612	26.49 (5.23)	1084	27.24 (5.81)	< 0.01
Waist circumference (cm)	3610	84.27 (13.31)	1085	86.01 (12.16)	< 0.01
Systolic blood pressure (mm/Hg)	3558	118.33 (12.49)	1076	118.17 (12.57)	0.72
Diastolic blood pressure (mm/Hg)	3558	72.52 (10.18)	1076	72.81 (11.16)	0.43
Glucose (mmol/dl)	3219	5.27 (0.89)	888	5.37 (1.24)	< 0.01
Insulin (u/ml)*	3202	4.58 (3.21, 6.7)	888	4.91 (3.42, 7.53)	< 0.01
Triglycerides (mmol/l)*	3218	0.88 (0.67, 1.18)	888	0.90 (0.69, 1.23)	0.05
LDL cholesterol (mmol/l)	3218	2.99 (0.80)	888	2.96 (0.83)	0.48
HDL cholesterol (mmol/l)	3218	1.49 (0.39)	888	1.44 (0.38)	< 0.01
CRP (mmol/l)*	3217	2.10 (3.59)	888	2.68 (4.46)	< 0.01
cIMT (mm)	3493	0.56 (0.06)	1050	0.55 (0.06)	0.09
Arterial distensibility (mm)	3485	0.50 (0.12)	1049	0.50 (0.12)	0.73
Covariables					
Age at outcome assessment	3612	48.13 (4.35)	1105	47.12 (4.82)	< 0.01
Smoking during adulthood					
Current smoker	3612	10.4	9302	23.3	< 0.01
Ex-smoker		9.4		15.5	
Never smoked		80.2		61.3	
Ethnicity					
White	3612	98.2	8636	97.1	< 0.01
Non-white		1.8		2.9	
Father’s (childhood) SEP					
Non-manual	3612	55.5	5890	42.7	< 0.01
Manual		46.5		57.3	
Adulthood SEP					
High	3612	66.2	7808	49.9	< 0.01
Low		33.8		50.1	

SD – standard deviation. IQR – interquartile range. BMI – body mass index. LDL- low density lipoprotein. HDL – high density lipoprotein. CRP – C-reactive protein. cIMT – carotid intima-media thickness.

* Median and interquartile range are presented for non-normally distributed variables.

Distributions of latent psychosocial adversity scores are not given for excluded participants because they were only modelled those participants eligible to be included in the analysis. Psychosocial exposures, ethnicity and childhood and adulthood SEP are given as percentages.

**Table 2:** Associations of cumulative psychosocial adversity in childhood and CVD risk factors at mean age 48 years (n = 3612).

	Adjusted for age		Adjusted for age, ethnicity and childhood SEP		Adjusted for age, ethnicity and childhood SEP and potential mediation by adult SEP		Adjusted for age, ethnicity and childhood SEP and potential mediation by adult SEP, BMI and smoking	
			
	Mean difference (95% CI)	P	Mean difference (95% CI)	P	Mean difference (95% CI)	P	Mean difference (95% CI)	P
BMI (kg/m^2^)	0.16 (−0.05, 0.37)	0.13	0.13 (−0.08, 0.34)	0.22	0.12 (−0.08, 0.33)	0.24	0.11 (−0.1, 0.32) †	0.30
Waist circumference (cm)	0.60 (0.10, 1.09)	0.02	0.53 (0.03, 1.02)	0.04	0.51 (0.02, 1.00)	0.04	0.39 (0.25, 1.57) †	0.12
SBP (mm/Hg)	−0.37 (−0.87, 0.13)	0.15	−0.40 (−0.90, 0.10)	0.12	−0.41 (−0.91, 0.09)	0.11	−0.46 (−0.94, 0.01)	0.06
DBP (mm/Hg)	−0.06 (−0.47, 0.35)	0.79	−0.10 (−0.51, 0.30)	0.62	−0.11 (−0.51, 0.30)	0.60	−0.18 (−0.56, 0.21)	0.37
Insulin (u/ml)*	−1% (−3%, 2%)	0.81	−1% (−3%, 2%)	0.66	−1% (−3%, 2%)	0.63	−1% (−4%, 1%)	0.17
Glucose (mmol/l)	0.01 (−0.03, 0.04)	0.74	0.01 (−0.03, 0.04)	0.77	0.01 (−0.03, 0.04)	0.78	−0.01 (−0.04, 0.03)	0.68
Triglycerides (mmol/l)*	1% (−1%, 2%)	0.59	1% (−1%, 2%)	0.63	0% (−1%, 2%)	0.66	−1% (−3%, 1%)	0.34
HDL-c (mmol/l)	−0.01 (−0.02, 0.01)	0.52	−0.01 (−0.02, 0.01)	0.62	−0.01 (−0.02, 0.01)	0.65	−0.01 (−0.01, 0.02)	0.63
LDL-c (mmol/l)	0.03 (−0.01, 0.06)	0.07	0.03 (−0.01, 0.06)	0.10	0.03 (−0.01, 0.06)	0.11	0.01 (−0.02, 0.05)	0.40
CRP (mmol/l)*	−1% (−5%, 5%)	0.94	−1% (−5%, 4%)	0.80	−1% (−5%, 4%)	0.76	−2% (−4%, 1%)	0.22
Arterial distensibility (mm)	−0.01 (−0.01, 0.004)	< 0.01	−0.01 (−0.01, −0.004)	< 0.01	−0.01 (−0.01, −0.004)	< 0.01	−0.01 (−0.01, −0.003)	< 0.01
cIMT (mm)	0.001 (−0.001, 0.003)	0.55	0.001 (−0.001, 0.003)	0.49	0.001 (−0.001, 0.003)	0.49	0.001 (−0.001, 0.003)	0.68

SEP – socioeconomic position. BMI-body mass index. SBP – systolic blood pressure. DBP – diastolic blood pressure. HDL-c – high density lipoprotein cholesterol. LDL-c – low density lipoprotein cholesterol. CRP – C reactive protein. CIMT – carotid intima-media thickness.

Coefficients are interpreted as the mean difference in the outcome per one standard deviation increase in the cumulative psychosocial adversity score.

* Coefficients for insulin, triglycerides and CRP have been back transformed from the natural log to a ratio of geometric means and are presented as the mean percentage difference in the outcome per one standard deviation increase in the cumulative psychosocial adversity score.

† Associations of cumulative adversity with BMI and waist circumference are adjusted for age, ethnicity, childhood SEP and potential mediation by adult SEP and smoking (not adult BMI).

## Appendix A. Supplementary data

Supplementary data associated with this article can be found, in the online version, at http://dx.doi.org/10.1016/j.chiabu.2017.10.015.
