# Supplementary material for: Associations of adversity in childhood and risk factors for cardiovascular disease in mid-adulthood
Source: Child Abuse Negl. Author manuscript; Available in PMC 2018 Apr 24. (PMC5915314; doi:10.1016/j.chiabu.2017.10.015)
Supplement: Supplement [file NIHMS959324-supplement-Supplement.docx]

**Online supplement**

**Assessment of cardiovascular risk factors**

Weight and height were measured with the subjects in light clothing and without shoes. Weight was measured to the nearest 0.1 kg with the use of Tanita scales. Height was measured to the nearest 0.1 cm with a Harpenden stadiometer. Body mass index (BMI) was calculated as weight in kilograms divided by height in meters-squared. Waist circumference was measured twice to the nearest 1mm at the midpoint between the lower ribs and the iliac crest with a flexible tape. The mean of the 2 measures was used in analyses. Blood pressure was measured while women were lying down with the use of an Omron M6 monitor (Omron Healthcare UK Ltd, Milton Keynes, UK). Two readings of systolic and diastolic blood pressure were recorded on each arm and the mean of these 4 readings was used in analyses. Women were asked to fast for a minimum of 8 hours before attending the assessment clinic. Blood samples were obtained, centrifuged, separated, and frozen at −80°C within 30 minutes. Plasma insulin was measured by an enzyme-linked immunosorbent assay (Mercodia, Uppsala, Sweden) that does not cross-react with proinsulin, and proinsulin was also measured by an enzyme-linked immunosorbent assay (Mercodia) that is a solid-phase 2-site enzyme immunoassay for the quantification of human proinsulin. The coefficients of variation are as follows: within assay, 3.2%; between assay, 5.2%; and total assay, 6.1%. There is no cross-reactivity with insulin or C-peptide. Plasma glucose was measured by automated enzymatic (hexokinase) method with coefficient of variation of <3%. Lipids were measured by automated analyser with enzymatic methods. C-reactive protein was measured by automated particle-enhanced immunoturbidimetric assay (Roche UK, Welwyn Garden City, UK). Left and right common carotid artery scans were obtained via high-resolution B ultrasound and imaged longitudinally 1cm proximal to the carotid bifurcation following a standardized protocol using a ZONARE z.one ultra-convertible ultrasound system with L10-5 linear transducer. Three consecutive cardiac cycles were identified and three measures of carotid intima-media thickness (cIMT) were taken from end-diastolic frames and averaged. This was done for both right and left carotid arteries and mean of the two was used in analyses. Arterial distensibility was calculated as the difference between systolic and diastolic arterial diameter in millimetres.

**Statistical analysis
*Confirmatory factor analyses***

Factor analyses were conducted using Mplus version 7.31 (Muthén & Muthén, 2008). Confirmatory first order factor analyses were used to estimate continuous latent constructs from responses to multiple questions (detailed in Table S1 below) about maternal bonding, parental mental illness, maladaptive family functioning, sexual and non-sexual abuse. Table S1 details factor loadings for each question asked (i.e. how well each variable loaded onto the factor) with larger numbers representing a better loading. Any value above 0.4 was considered to be an acceptable factor loading, although this value is arbitrary. The majority of variables loaded onto their corresponding first order factors very well. To assess the fit of each first order factor model, three fit statistics were used: (i) root-mean-square error of approximations (RMSEA)(1); (ii) comparative fit index (CFI)(2); and (iii) Tucker– Lewis fit index (TLI).(3) Table S1 below details the model fit statistics for each factor. The first order factors fit well. The ‘maternal overprotection’ factor had slightly lower CFI and TLI values than the other factors, however, the factor loading of each variable was large and the RMSEA was reasonable.

***Cumulative psychosocial adversity in childhood***We conducted a second order factor analysis model which captures all of the individual latent psychosocial adversity constructs listed above. Adverse experiences that are more strongly correlated with other adverse experiences (and therefore experiences that more likely co-occur) are assigned higher factor loadings, and therefore contribute more to the factor score. Table S1 details the factor loadings for each of the continuous latent constructs onto the second order factor. Latent constructs loaded onto the second order factor well. As above, RMSEA, CFI and TLI were used to assess model fit (Table S1 below). Model fit was good, especially given the complexity of the model.

**Table S1. Prevalence and model fit for psychosocial adversity factors**

| **LATENT ADVERSITY CONSTRUCT**  Exact questions asked in questionnaires | **% Prevalence in included (n=3,612)*** | **% Prevalence in excluded (n=10,929)**** | **P for difference***** | **Factor loadings for each variable** | **Model fit statistics for each latent construct** |
| --- | --- | --- | --- | --- | --- |
| **First order factors** | | | | | |
| **MATERNAL LACK OF CARE** |  |  |  |  |  |
| Did your mother speak to you in a warm and friendly voice? (RS) | 16.6 | 22.9 | <0.01 | 0.89 | RMSEA = 0.06  CFI =0.98 TLI = 0.98 |
| Did your mother help you as much as you needed? (RS) | 16.8 | 20.9 | <0.01 | 0.90 |  |
| Did your mother seem emotionally cold to you? | 4.4 | 5.1 | 0.10 | 0.73 |  |
| Problems understood by the mother (RS) | 48.7 | 49.8 | 0.30 | 0.83 |  |
| Was your mother affectionate towards you? (RS) | 26.4 | 31.2 | <0.01 | 0.87 |  |
| Did your mother make you feel you were not wanted? | 2.5 | 4.5 | <0.01 | 0.62 |  |
| Did your mother talk things over with you? (RS) | 56.1 | 55.3 | 0.45 | 0.82 |  |
| Did your mother praise you? (RS) | 43.9 | 49.5 | <0.01 | 0.81 |  |
| Did your mother enjoy talking things over with you? (RS) | 18.5 | 19.9 | 0.08 | 0.87 |  |
| Did your mother frequently smile at you? (RS) | 9.5 | 10.8 | 0.03 | 0.90 |  |
| Did your mother seem to understand what you needed or wanted? (RS) | 21.9 | 22.2 | 0.70 | 0.90 |  |
| Did your mother make you feel better when you were upset? (RS) | 12.3 | 14.3 | <0.01 | 0.88 |  |
| **MATERNAL OVERPROTECTION** |  |  |  |  |  |
| Did your mother allow you to things you liked doing? (RS) | 37.5 | 44.1 | <0.01 | 0.64 | RMSEA = 0.08  CFI =0.96 TLI = 0.93 |
| Did your mother try to control what you did? | 23.2 | 26.3 | <0.01 | 0.53 |  |
| Did your mother let you decide things for yourself? (RS) | 51.8 | 56.1 | <0.01 | 0.71 |  |
| Did your mother give you the freedom you wanted? (RS) | 61.3 | 65.5 | <0.01 | 0.84 |  |
| Did your mother let you go out as often as you wanted? (RS) | 56.5 | 53.5 | <0.01 | 0.79 |  |
| Was your mother overprotective of you? | 20.0 | 21.4 | 0.08 | 0.52 |  |
| Did your mother allow you to dress in any way you pleased? (RS) | 45.6 | 41.1 | <0.01 | 0.71 |  |
| **MALADAPTIVE FAMILY FUNCTIONING** |  |  |  |  |  |
| Was your parent's behaviour stable and predictable to you as a child? (RS) | 10.3 | 13.4 | <0.01 | 0.77 | RMSEA = 0.06  CFI =0.98 TLI = 0.97 |
| Did your parents have serious arguments? | 25.7 | 27.3 | 0.07 | 0.80 |  |
| Was your parent’s relationship violent? | 10.6 | 15.2 | <0.01 | 0.89 |  |
| Was your parent’s relationship affectionate? (RS) | 11.0 | 13.4 | <0.01 | 0.80 |  |
| Was your parent’s relationship quarrelsome? | 75.8 | 78.7 | <0.01 | 0.65 |  |
| Was your parent’s relationship happy? (RS) | 4.1 | 5.7 | <0.01 | 0.95 |  |
| Was your parent’s relationship frightening? | 17.1 | 20.5 | <0.01 | 0.87 |  |
| Was your parent’s relationship friendly? (RS) | 2.9 | 4.2 | <0.01 | 0.93 |  |
| Was your parent’s relationship respectful? (RS) | 6.6 | 10.2 | <0.01 | 0.90 |  |
| Was your parent’s relationship remote or distant? | 41.3 | 42.7 | 0.21 | 0.75 |  |
| Parental separation | 11.5 | 19.4 | <0.01 | 0.70 |  |
| **PARENTAL MENTAL ILLNESS** |  |  |  |  |  |
| Was either parent mentally ill before age 17 years? | 4.4 | 4.1 | 0.43 | 0.74 | RMSEA = 0.06  CFI =0.96 TLI = 0.87 |
| Did your mother have depression or nerves? | 20.1 | 22.7 | <0.01 | 0.61 |  |
| Did your mother have an alcohol problem? | 2.1 | 3.0 | 0.01 | 0.88 |  |
| Did your father have an alcohol problem? | 5.4 | 6.9 | <0.01 | 0.33 |  |
| **SEXUAL ABUSE** |  |  |  |  |  |
| Unwanted or abusive masturbation^a^ | 3.4 | 4.3 | 0.02 | 0.73 | RMSEA = 0.03  CFI =0.99 TLI = 0.98 |
| Unwanted or abusive fondling^a^ | 9.6 | 10.2 | 0.35 | 0.86 |  |
| Unwanted or abusive genital rubbing^a^ | 5.2 | 6.1 | 0.08 | 0.92 |  |
| Unwanted or abusive oral sex^a^ | 1.0 | 2.3 | <0.01 | 0.80 |  |
| Unwanted or abusive intercourse^a^ | 1.2 | 2.6 | <0.01 | 0.88 |  |
| **NON-SEXUAL ABUSE** |  |  |  |  |  |
| Parent was physically cruel | 2.7 | 3.7 | <0.01 | 0.82 | RMSEA = 0.04  CFI =0.99 TLI = 0.98 |
| Parent was emotionally cruel | 7.0 | 7.9 | 0.11 | 0.97 |  |
| Physically neglected by parent | 1.1 | 2.5 | <0.01 | 0.73 |  |
| Emotionally neglected by parent | 18.7 | 23.5 | <0.01 | 0.75 |  |
| **Second order factor** | | | | | |
| **CUMULATIVE PSYCHOSICIAL ADVERSITY** |  |  |  |  | RMSEA = 0.03  CFI =0.95 TLI =0.94 |
| Lack of care factor | - | - | - | 0.77 |  |
| Overprotection factor | - | - | - | 0.51 |  |
| Maladaptive family functioning factor | - | - | - | 0.71 |  |
| Parental mental illness factor | - | - | - | 0.63 |  |
| Sexual abuse factor | - | - | - | 0.41 |  |
| Non-sexual abuse factor | - | - | - | 0.99 |  |

RS=Reverse Score. All prevalence estimates are given for the ‘Yes’ category, except when RS is indicated, where prevalence is given for the ‘No’ category.
^a^Multiple questions were asked about sexual experiences (including unwanted experiences) before age 16. Questions were asked in relation to the type of experience, who was involved in the experience, whether or not the participant wanted it to happen, how old they were when it first happened and how often it happened, and from responses to these questions the abuse variables were derived by the ALSPAC data team.
*Prevalence of exposure in participants included in the analysis
**Prevalence of exposure in participants excluded from the analysis due to missing data for the adversity exposures, the outcomes or the potential confounders and mediators
***P values for differences in prevalence estimates between included and excluded participants were obtained from a chi-squared test.

**Table S2: Predictors of inclusion in main analysis sample (n=3612) of women with complete data for potential confounders, from the larger sample of women (n=4541) with incomplete data for potential confounders**

| **Predictor of inclusion on main analysis sample of n=3612** | **Larger sample of women with data for at least one adversity exposure and one CVD risk factor (n=4541)** | |
| --- | --- | --- |
|  | N | % |
| **Maternal education**  < O-level  O level  A level  Degree or above | 4447  (2% missing) | 17.9  34.9  28.7  18.5 |
| **Paternal education**  < O-level  O level  A level  Degree or above | 4345 (4% missing) | 24.5 21.2 29.3  25.0 |
| **Patent died before age 17** No  Yes | 4424 (3% missing) | 94.0  6.0 |
| **Parent absent before age 17** No Yes | 4446 (2% missing) | 82.7 17.3 |
| **Ethnicity** White Non-White | 4433  (2% missing) | 97.7  2.3 |
| **Experienced apparent coldness from mother before age 17**  No Yes | 4394  (3% missing) | 95.5  4.4 |
| **Allowed to make own decisions by mother before age 17**  Yes No | 4391 (3% missing) | 48.2  51.8 |
| **Legally adopted**  No Yes | 4418 (3% missing) | 97.5  2.5 |
| **Experienced physical abuse as a child** No Yes | 4089  (10% missing) | 94.6  5.4 |

SD-Standard Deviation

**Table S3. Distribution of adverse psychosocial experiences by childhood SEP (n=3612)**

|  | **Non-manual childhood SEP (n=2392)** | **Manual childhood SEP (n=1220)** |  |
| --- | --- | --- | --- |
| **Continuous factor variables** | **Mean (SD)** | **Mean (SD)** | **P for difference** |
| Parental bonding - lack of care | 0.06 (0.80) | 0.10 (0.85) | 0.20 |
| Parental bonding - overprotection | 0.02 (0.77) | -0.02 (0.80) | 0.14 |
| Maladaptive family functioning | 0.06 (0.76) | 0.13 (0.81) | 0.01 |
| Parental mental illness | 0.13 (0.54) | 0.16 (0.55) | 0.72 |
| Sexual abuse | 0.17 (0.52) | 0.17 (0.55) | 0.86 |
| Non-sexual abuse | 0.13 (0.57) | 0.15 (0.60) | 0.31 |

SEP – socioeconomic position. Distribution in the continuous factor variables is mean (SD) of the factor scores and P for difference was obtained using a two-tailed t-test.

**Table S4. Associations of individual types of psychosocial adversity with cardiovascular risk factors. N=3612 for all types of adversity. Continued on following page)**

|  | **Lack of care** | |  | **Overprotection** | |  | **Maladaptive family functioning** | |
| --- | --- | --- | --- | --- | --- | --- | --- | --- |
|  | **B (95% CI)** | **P** |  | **B (95% CI)** | **P** |  | **B (95% CI)** | **P** |
| BMI (kg/m^2^) | 0.16 (-0.03, 0.36) | 0.10 |  | 0.12 (-0.07, 0.32) | 0.18 |  | -0.07 (-0.28, 0.14) | 0.52 |
| Waist circumference (cm) | 0.60 (0.14, 1.05) | 0.01 |  | 0.43 (-0.01, 0.88) | 0.05 |  | -0.06 (-0.56, 0.44) | 0.81 |
| SBP (mm/Hg) | -0.32 (-0.80, 0.15) | 0.19 |  | -0.01 (-0.48, 0.46) | 0.99 |  | -0.40 (-0.91, 0.11) | 0.12 |
| DBP (mm/Hg) | -0.03 (-0.40, 0.35) | 0.90 |  | -0.02 (-0.40, 0.36) | 0.94 |  | -0.29 (-0.70, 0.13) | 0.18 |
| Insulin (u/ml)* | 1.03 (1.00, 1.06) | 0.06 |  | 1.00 (0.97, 1.03) | 0.97 |  | 0.99 (0.96, 1.01) | 0.38 |
| Glucose (mmol/l) | -0.01 (-0.06, 0.03) | 0.63 |  | 0.04 (-0.01, 0.08) | 0.09 |  | -0.01 (-0.04, 0.03) | 0.76 |
| Triglycerides (mmol/l)* | 1.00 (0.98, 1.02) | 0.91 |  | 1.00 (0.97, 1.02) | 0.89 |  | 1.00 (0.99, 1.02) | 0.68 |
| HDL-c (mmol/l) | 0.004 (-0.03, 0.03) | 0.94 |  | 0.004 (-0.03, 0.04) | 0.84 |  | 0.01 (-0.01, 0.02) | 0.44 |
| LDL-c (mmol/l) | 0.01 (-0.01, 0.02) | 0.60 |  | -0.003 (-0.02, 0.01) | 0.78 |  | 0.02 (-0.01, 0.05) | 0.24 |
| CRP (mmol/l)* | 0.99 (0.97, 1.00) | 0.08 |  | 1.00 (0.98, 1.01) | 0.53 |  | 0.99 (0.94 1.04) | 0.65 |
| Arterial distensibility (mm) | -0.01 (-0.01, 0.00) | 0.02 |  | -0.004 (-0.01, -0.0001) | 0.07 |  | -0.004 (-0.01, -0.0001) | 0.13 |
| CIMT (mm) | 0.001 (-0.001, 0.003) | 0.41 |  | 0.002 (0.0001, 0.004) | 0.07 |  | -0.001 (-0.003, 0.001) | 0.66 |

BMI-body mass index. SBP – systolic blood pressure. DBP – diastolic blood pressure. HDL-c – high density lipoprotein cholesterol. LDL-c - low density lipoprotein cholesterol. CRP – C reactive protein. CIMT – carotid intima-media thickness.
*Coefficients for insulin, triglycerides and CRP have been back transformed from the natural log and can be interpreted as a ratio of geometric means, (e.g. a coefficient of 1.02 would be interpreted as an average of 2% increase in the outcome per standard deviation increase in cumulative psychosocial adversity)

**Table S4 continued.**

|  |  | **Parental mental illness** | | **Sexual abuse** | |  | **Non-sexual abuse** | |
| --- | --- | --- | --- | --- | --- | --- | --- | --- |
|  |  | **B (95% CI)** | **P** | **B (95% CI)** | **P** |  | **B (95% CI)** | **P** |
| BMI (kg/m^2^) |  | 2.66 (-18.1, 23.41) | 0.80 | -0.03 (-0.35, 0.28) | 0.83 |  | 0.16 (-0.12, 0.43) | 0.26 |
| Waist circumference (cm) |  | 7.6 (-51.93, 67.14) | 0.80 | 0.11 (-0.62, 0.84) | 0.77 |  | 0.54 (-0.11, 1.19) | 0.11 |
| SBP (mm/Hg) |  | 8.49 (-58.73, 75.71) | 0.81 | -0.08 (-0.79, 0.63) | 0.82 |  | -0.36 (-1.03, 0.31) | 0.29 |
| DBP (mm/Hg) |  | 6.43 (-44.41, 57.28) | 0.80 | 0.06 (-0.53, 0.64) | 0.85 |  | -0.02 (-0.56, 0.52) | 0.94 |
| Insulin (u/ml)* |  | 1.00 (0.97, 1.03) | 0.94 | 0.99 (0.96, 1.03) | 0.71 |  | 1.00 (0.97, 1.04) | 0.96 |
| Glucose (mmol/l) |  | -0.02 (-0.07, 0.03) | 0.38 | 0.02 (-0.04, 0.07) | 0.56 |  | 0.03 (-0.02, 0.07) | 0.31 |
| Triglycerides (mmol/l)* |  | 1.01 (0.99, 1.03) | 0.32 | 1.02 (1.00, 1.05) | 0.10 |  | 1.00 (0.97, 1.03) | 0.99 |
| HDL-c (mmol/l) |  | 0.02 (0, 0.04) | 0.13 | 0.01 (-0.01, 0.03) | 0.34 |  | 0.01 (-0.01, 0.03) | 0.33 |
| LDL-c (mmol/l) |  | 0.001 (-0.01, 0.01) | 0.55 | 0.01 (-0.04, 0.06) | 0.66 |  | 0.03 (-0.02, 0.08) | 0.18 |
| CRP (mmol/l)* |  | 1.00 (1.00, 1.00) | 0.83 | 1.00 (0.93, 1.08) | 0.97 |  | 0.98 (0.92, 1.04) | 0.48 |
| Arterial distensibility (mm) |  | 0.01 (-0.03, 0.03) | 0.94 | -0.01 (-0.01, 0.002) | 0.13 |  | -0.01 (-0.01, -0.002) | 0.01 |
| CIMT (mm) |  | -0.02 (-0.07, 0.03) | 0.38 | -0.001 (-0.005, 0.003) | 0.50 |  | 0.001 (-0.003, 0.005) | 0.66 |

**Table S5. Associations of psychosocial adversity with cardiovascular risk factors by high and low childhood SEP**

|  | **High adult SEP (n=2,392)** | **Low adult SEP (n=1220)** |  |
| --- | --- | --- | --- |
|  | **Adjusted for age, ethnicity and childhood SEP** | | |
|  | **B (95%, CI)** | **B, (95%, CI)** | **P for interaction** |
| BMI (kg/m^2^) | 0.21 (0.01, 0.42) | -0.14 (-0.46, 0.17) | 0.04 |
| Waist circumference (cm) | 0.65 (0.18, 1.12) | -0.13 (-0.86, 0.60) | 0.06 |
| SBP (mm/Hg) | -0.24 (-0.74, 0.26) | -0.35 (-1.05, 0.35) | 0.84 |
| DBP (mm/Hg) | -0.11 (-0.52, 0.29) | 0.09 (-0.51, 0.68) | 0.59 |
| Insulin (u/ml)* | 0.99 (0.98, 1.03) | 0.97 (0.93, 1.00) | 0.10 |
| Glucose (mmol/l) | 0.02 (-0.02, 0.06) | -0.01 (-0.07, 0.04) | 0.33 |
| Triglycerides (mmol/l)* | 1.00 (0.99, 1.02) | 1.00 (0.98, 1.03) | 0.92 |
| HDL-c (mmol/l) | 0.003 (-0.01, 0.02) | 0.005 (-0.02, 0.03) | 0.83 |
| LDL-c (mmol/l) | 0.01 (-0.02, 0.04) | 0.04 (-0.01, 0.09) | 0.36 |
| CRP (mmol/l)* | 1.02 (0.98, 1.07) | 1.00 (0.88, 1.01) | 0.04 |
| Arterial distensibility (mm) | -0.005 (-0.009, -0.0002) | -0.003 (-0.009, 0.004), | 0.57 |
| CIMT (mm) | -0.00003 (-0.002, 0.002) | 0.0008 (-0.003, 0.004), | 0.76 |

SEP – socioeconomic position. BMI-body mass index. SBP – systolic blood pressure. DBP – diastolic blood pressure. HDL-c – high density lipoprotein cholesterol. LDL-c - low density lipoprotein cholesterol. CRP – C reactive protein. CIMT – carotid intima-media thickness.
*Coefficients for insulin, triglycerides and CRP have been back transformed from the natural log and can be interpreted as a ratio of geometric means, (e.g. a coefficient of 1.02 would be interpreted as an average of 2% increase in the outcome per standard deviation increase in cumulative psychosocial adversity)

**Table S6. Associations of cumulative psychosocial adversity in childhood and CVD risk factors after additional adjustment for medication use (N=3612)**

|  | **Adjusted for age, ethnicity childhood SEP and medication use** | |
| --- | --- | --- |
|  | **B (95% CI)** | **P** |
| SBP (mm/Hg) | -0.43 (-0.92, 0.06) | 0.08 |
| DBP (mm/Hg) | -0.12 (-0.52, 0.28) | 0.55 |
| Insulin (u/ml)* | 0.99 (0.97, 1.02) | 0.67 |
| Glucose (mmol/l) | 0.01 (-0.03, 0.04) | 0.75 |
| Triglycerides (mmol/l)* | 1.01 (0.99, 1.02) | 0.53 |
| HDL-c (mmol/l) | -0.01 (-0.02, 0.01) | 0.52 |
| LDL-c (mmol/l) | 0.03 (-0.01, 0.06) | 0.15 |

SEP – socioeconomic position. SBP – systolic blood pressure. DBP – diastolic blood pressure. HDL-c – high density lipoprotein cholesterol. LDL-c - low density lipoprotein cholesterol. Where SBP and DBP are the outcomes, associations are adjusted for use of antihypertensive medication. Where insulin and glucose are the outcomes, associations are adjusted for diabetes medication use. When triglycerides, HDL and LDL are the outcomes, associations are adjusted for stain use.
*Coefficients for insulin and triglycerides have been back transformed from the natural log and can be interpreted as a ratio of geometric means, (e.g. a coefficient of 1.02 would be interpreted as an average of 2% increase in the outcome per standard deviation increase in cumulative psychosocial adversity)

**Table S7. Associations of cumulative psychosocial adversity in childhood and CVD risk factors after removing participants who reported medication use at the time of outcome assessment (i.e. reported taking any antihypertensive, diabetes or statin medication)**

|  |  | **Adjusted for age, ethnicity childhood SEP and medication use** | |  |
| --- | --- | --- | --- | --- |
|  | **N** | **B (95% CI)** | **P** |  |
| SBP (mm/Hg) | 3255 | -0.44 (-0.93, 0.06) | 0.08 |  |
| DBP (mm/Hg) | 3255 | -0.10 (-0.51, 0.31) | 0.64 |  |
| Insulin (u/ml)* | 3408 | 0.99 (0.97, 1.02) | 0.58 |  |
| Glucose (mmol/l) | 3408 | -0.01 (-0.04, 0.03) | 0.76 |  |
| Triglycerides (mmol/l)* | 3372 | 1.01 (0.99, 1.02) | 0.60 |  |
| HDL-c (mmol/l) | 3372 | -0.01 (-0.02, 0.01) | 0.57 |  |
| LDL-c (mmol/l) | 3372 | 0.02 (-0.01, 0.06) | 0.16 |  |

SEP – socioeconomic position. SBP – systolic blood pressure. DBP – diastolic blood pressure. HDL-c – high density lipoprotein cholesterol. LDL-c - low density lipoprotein cholesterol.
*Coefficients for insulin and triglycerides have been back transformed from the natural log and can be interpreted as a ratio of geometric means, (e.g. a coefficient of 1.02 would be interpreted as an average of 2% increase in the outcome standard deviation increase in cumulative psychosocial adversity)

**Table S8. Associations of cumulative psychosocial adversity in childhood and CVD risk factors in complete case samples (i.e. with no missing data, n=2101)**

|  | **Adjusted for age, ethnicity and childhood SEP** | |
| --- | --- | --- |
|  | **B (95% CI)** | **P** |
| BMI (kg/m^2^) | 0.07 (-0.19, 0.33) | 0.61 |
| Waist circumference (cm) | 0.44 (-0.2, 1.07) | 0.18 |
| SBP (mm/Hg) | -0.53 (-1.18, 0.12) | 0.11 |
| DBP (mm/Hg) | -0.25 (-0.77, 0.26) | 0.33 |
| Insulin (u/ml)* | 1.01 (0.97, 1.04) | 0.77 |
| Glucose (mmol/l) | 0.01 (-0.04, 0.06) | 0.65 |
| Triglycerides (mmol/l)* | 1.00 (0.98, 1.02) | 0.83 |
| HDL-c (mmol/l) | -0.002 (-0.02, 0.02) | 0.85 |
| LDL-c (mmol/l) | 0.03 (-0.01, 0.07) | 0.12 |
| CRP (mmol/l)* | 0.99 (0.94, 1.05) | 0.88 |
| Arterial distensibility (mm) | -0.01 (-0.01, -0.0001) | 0.05 |
| CIMT (mm) | -0.001 (-0.005, 0.003) | 0.54 |

SEP – socioeconomic position. BMI-body mass index. SBP – systolic blood pressure. DBP – diastolic blood pressure. HDL-c – high density lipoprotein cholesterol. LDL-c - low density lipoprotein cholesterol. CRP – C reactive protein. CIMT – carotid intima-media thickness.
*Coefficients for insulin, triglycerides and CRP have been back transformed from the natural log and can be interpreted as a ratio of geometric means, (e.g. a coefficient of 1.02 would be interpreted as an average of 2% increase in the outcome per standard deviation increase in cumulative psychosocial adversity)

**Table S9. Associations of cumulative psychosocial adversity in childhood and CVD risk factors, using inverse probability weightings to weight regression analyses on our main analysis sample (n=3612) of women with complete confounder data, compared to the larger sample of women (n=4541) of women with incomplete confounder data**

|  | **Adjusted for age, ethnicity and childhood SEP** | |
| --- | --- | --- |
|  | **B (95% CI)** | **P** |
| BMI (kg/m^2^) | 0.12 (-0.09, 0.33) | 0.25 |
| Waist circumference (cm) | 0.51 (0.01, 1.01) | 0.04 |
| SBP (mm/Hg) | -0.41 (-0.91, 0.09) | 0.11 |
| DBP (mm/Hg) | -0.11 (-0.51, 0.3) | 0.61 |
| Insulin (u/ml)* | 0.99 (0.97, 1.02) | 0.63 |
| Glucose (mmol/l) | 0.01 (-0.03, 0.04) | 0.78 |
| Triglycerides (mmol/l)* | 1.00 (0.99, 1.02) | 0.66 |
| HDL-c (mmol/l) | -0.003 (-0.02, 0.01) | 0.68 |
| LDL-c (mmol/l) | 0.03 (-0.01, 0.06) | 0.11 |
| CRP (mmol/l)* | 0.99 (0.94, 1.04) | 0.75 |
| Arterial distensibility (mm) | -0.01 (-0.01, -0.003) | 0.01 |
| CIMT (mm) | 0.001 (-0.001, 0.003) | 0.49 |

SEP – socioeconomic position. BMI-body mass index. SBP – systolic blood pressure. DBP – diastolic blood pressure. HDL-c – high density lipoprotein cholesterol. LDL-c - low density lipoprotein cholesterol. CRP – C reactive protein. CIMT – carotid intima-media thickness.
*Coefficients for insulin, triglycerides and CRP have been back transformed from the natural log and can be interpreted as a ratio of geometric means, (e.g. a coefficient of 1.02 would be interpreted as an average of 2% increase in the outcome per standard deviation increase in cumulative psychosocial adversity)a

**Table S10. Comparing associations of cumulative psychosocial adversity with pre-pregnancy BMI at mean age 29 years (n=8,517) with BMI at mean age 51 years (n=3,612) to assess the possibility of selection bias**

|  | **Pre-pregnancy BMI at mean age 29 years** | |  | **BMI at mean age 51 years** | |  |
| --- | --- | --- | --- | --- | --- | --- |
|  | **B (95% CI)** | **P** |  | **B (95% CI)** | **P** |  |
| BMI (kg/m^2^) | 0.13 (-0.08, 0.34) | 0.22 |  | 0.02 (-0.08, 0.12) | 0.68 |  |

BMI-body mass index. SBP – systolic blood pressure. DBP – diastolic blood pressure. HDL-c – high density lipoprotein cholesterol. LDL-c - low density lipoprotein cholesterol. CRP – C reactive protein. CIMT – carotid intima-media thickness.
*Coefficients for insulin, triglycerides and CRP have been back transformed from the natural log and can be interpreted as a ratio of geometric means, (e.g. a coefficient of 1.02 would be interpreted as an average of 2% increase in the outcome per standard deviation increase in cumulative psychosocial adversity)

**Table S11. Associations of cumulative psychosocial adversity in childhood and CVD risk factors, only in women who had reached menopause (n=890)**

|  | **Adjusted for age, ethnicity and childhood SEP** | |
| --- | --- | --- |
|  | **B (95% CI)** | **P** |
| BMI (kg/m^2^) | 0.41 (0.01, 0.8) | 0.04 |
| Waist circumference (cm) | 1.12 (0.17, 2.06) | 0.02 |
| SBP (mm/Hg) | -0.16 (-1.13, 0.82) | 0.75 |
| DBP (mm/Hg) | -0.10 (-0.92, 0.72) | 0.81 |
| Insulin (u/ml)* | 1.01 (0.96, 1.06) | 0.61 |
| Glucose (mmol/l) | 0.01 (-0.08, 0.09) | 0.89 |
| Triglycerides (mmol/l)* | 1.01 (0.97, 1.05) | 0.55 |
| HDL-c (mmol/l) | 0.001 (-0.04, 0.03) | 0.85 |
| LDL-c (mmol/l) | 0.04 (-0.03, 0.11) | 0.22 |
| CRP (mmol/l)* | 1.02 (0.93, 1.10) | 0.73 |
| Arterial distensibility (mm) | 0.01 (-0.02, -0.01) | <0.001 |
| CIMT (mm) | 0.001 (-0.01, 0.01) | 0.54 |

SEP – socioeconomic position. BMI-body mass index. SBP – systolic blood pressure. DBP – diastolic blood pressure. HDL-c – high density lipoprotein cholesterol. LDL-c - low density lipoprotein cholesterol. CRP – C reactive protein. CIMT – carotid intima-media thickness.
*Coefficients for insulin, triglycerides and CRP have been back transformed from the natural log and can be interpreted as a ratio of geometric means, (e.g. a coefficient of 1.02 would be interpreted as an average of 2% increase in the outcome per standard deviation increase in cumulative psychosocial adversity)

1. Steiger JL, JC. Statistically based tests for the number of common factors. Annual Spring Meeting of the Psychometric Society; Iowa City, IA1980.

2. Bentler PM. Comparative fit indexes in structural models. Psychol Bull. 1990;107(2):238-46.

3. Tucker LL, C. A reliability coefficient for maximum likelihood factor analysis. Psychometrika. 1973;38(1):1-10.
